# A parallel journey: developing an eating disorders program; perspectives from an acute medical ward

**DOI:** 10.1186/2050-2974-2-S1-O27

**Published:** 2014-11-24

**Authors:** Mary Bronson, Lisa Miller, Jan Fountaine, Charlotte Simmonds, Christina Bygrave, Keira James, Melissa Edwin, Anthea Fursland, Patrick Marwick

**Affiliations:** 1 Sir Charles Gairdner Hospital, Perth, Australia; 2 North Metro Area Mental Health Service, Perth, Australia; 3 Centre for Clinical Intervention, Perth, Australia

## 

From February 2012 a multidisciplinary group in a 600 bed tertiary public hospital in Western Australia have worked collaboratively to improve the care of adult patients admitted to an acute medical ward with complications of severe eating disorders

This clinical care journey has gone on to inform a program of staff development and strategic advocacy for a state wide approach to the care of adults with eating disorders.

Via demonstration of parallel journeys of patients, staff and the state wide strategy, the authors have identified areas of unmet need, gaps in resourcing and barriers to improving care from a local context.

This multidisciplinary collaborative process has now informed a business case for a comprehensive specialist eating disorders service for youths and adults in Western Australia's public health service.

This abstract was presented in the **Service Initiatives** stream of the 2014 ANZAED Conference.

